# Optimization of Cactus Pear Fruit Fermentation Process for Wine Production

**DOI:** 10.3390/foods7080121

**Published:** 2018-07-30

**Authors:** Zenebe Tadesse Tsegay, Chanukya Basavanahally Sathyanarayana, Solomon Mengistu Lemma

**Affiliations:** 1 College of Natural and Computational Science, Department of Chemistry, Aksum University, P.O. Box 1010, Aksum, Ethiopia; ztlovewith73@gmail.com; 2 Department of Chemical and Food Engineering, Bahir Dar Institute of Technology, Bahir Dar University, P.O. Box 26, Bahir Dar, Ethiopia; bs.chanu@gmail.com; 3 Golden LEAF Biomanufacturing Training and Education Center (BTEC), North Carolina State University, Raleigh, NC 27606, USA

**Keywords:** Cactus pear fruit, wine, fermentation, optimization, response surface methodology (RSM)

## Abstract

Cactus pear fruit (*Opuntia ficus-indica*) has a chemical composition that renders it an attractive substrate for wine fermentation. However, there have been serious post-harvest losses of cactus fruit due to its short shelf life. This study aims to investigate wine production from cactus pear fruit juice by optimizing fermentation temperature, pH, and inoculum concentration (*Saccharomyces cerevisiae*) to obtain optimum quality-indicative responses. Response surface methodology coupled with central composite rotatable design was adopted in the present study to achieve optimized fermentation process conditions. The fermentation process was carried out for 6 days with varied input variables, and all the models showed significant *p*-values for interaction of variance (<0.05). Cactus pear fruit wine with a total acidity of 12.39 ± 1.32 g/L equivalent to tartaric acid (TTAE), alcohol content of 9 ± 0.31%, *v/v*, total antioxidant concentration of 235.3 ± 9.15 mg/L AAE (Ascorbic acid equivalent), and sensory acceptance of 7.74 ± 0.34 was produced at an optimized temperature of 30 °C, pH of 3.9, and inoculum concentration of 16%. The developed models could predict the quality of wine developed from cactus pear fruit.

## 1. Introduction

Wine production is one of the oldest fermentation processes [1]. Specifically, wine from grapes has been studied and well documented for a long time. However, earlier researchers have conducted a number of studies in search of alternative fruits as raw materials for wine production [2]. Cactus pear fruit is one such suitable fruit source for the production of acceptable quality wine [3]. Cactus pear fruit is a good source of nutrients, such as lutein, lycopene, β-carotene, minerals, ascorbic acid, betalains, and indicaxanthin [4,5,6]. The presence of such chemical compositions in the fruit encourages the production of unique wines and other fermented beverages [3,7]. Limited studies have been carried out on the production of wine from cactus pear fruit within the last three decades [3,8,9]. Furthermore, this fruit is highly perishable and seasonal. Hence, developing a predictable fermentation process model is important and may benefit producers of cactus pear fruit worldwide. 

Since wine typically contains alcohol, polyphenol, and other chemical components (titratable and volatile organic acids) and has a pH range of 3.2–3.8, it can be preserved while retaining its good sensory qualities for more than a year [1,10]. It is well known that the fermentation process is affected by factors such as temperature, pH, nitrogen level, nutrients in the substrate, type of yeast strain, and ratio of inoculum to the substrate [3,11,12]. As a result, flavor, aroma, color, and alcohol content of wine products can vary from batch to batch. Hence, the initial set values of input variables directly influence the final quality of wine. Navarrete-Bolaños et al. reported the results of cactus pear fruit fermentation for wine production with optimized alcohol content, volatile compound profile, organic acid profile, and specific compounds related to color [3]. The report shows that the fermentation process can result in a significant development of volatile compounds, with better aroma and flavor of the produced wine. Response surface methodology (RSM) is a statistical tool used to optimize wine fermentation process parameters. It has been applied to control the parameters of fermenting mango, apple, and Kinnow mandarin fruit, all of which have chemical compositions similar to cactus fruit [2,11,13,14].

Considering the above factors, using response surface methodology (RSM) coupled with central composite rotatable design (CCRD), this study aims to optimize the three basic variables of cactus pear fruit fermentation process—that is, fermentation temperature, pH, and inoculum concentration—to control the quality attributes of wine, such as: total acidity, alcohol, antioxidant content, and sensory properties, using a strain of *Saccharomyces cerevisiae*. This paper further demonstrates and reports the values of the optimized process parameters, and the effect these parameters have on the fermentation process for acceptable wine production.

## 2. Materials and Methods 

### 2.1. Materials 

Ripe cactus pear fruit (variety: *Opuntia ficus-indica)* was obtained from farmers in Adigrat, Ethiopia, during peak the production time of March and April. Wine yeast extract of the *Saccharomyces cerevisiae* strain used in the study (Montrachet, UCD 522) was donated by Awash Wine Factory, Addis Ababa, Ethiopia. Sodium phosphate was purchased from UNI-CHEM, Mumbai, India. Ammonium molybdate, sodium molybdate, sodium carbonate, yeast extract, peptone water, d-glucose, phenol (pH value = 4.8–6.0), potassium dichromate, gallic acid, ascorbic acid, sodium hydroxide, tartaric acid, and sodium thiosulphate were purchased from Loba-Chemie Laboratory Reagents and Fine Chemicals Co. India, Mumbai, India. All chemicals and solvents used in this study were of analytical grade and used as supplied.

### 2.2. Cactus Pear Fruit Juice Extraction

Ripe cactus fruits (12.5 kg) were stored in an ice box at 6 °C during their 3 h transportation to Aksum University Chemistry Laboratory. The fruits were selected based on physical appearance, sorted, and gently washed in running tap water in order to remove all the spines. Subsequently, the fruits were peeled manually to remove their outer coat. The 9 kg edible part was chopped with seeds using a domestic juicer (Electric Juicer, BL–727, V.P Enterprise, Jaipur, India) and homogenized manually for 25 min at room temperature. The juice was filtered using sterilized cotton cloth mesh to remove the seed and fibers and was then preserved at 4 °C until usage. 

### 2.3. Cactus Pear Fruit Must Development

The physicochemical profile of the must before fermentation was characterized based on degree brix, pH, total acidity, antioxidant, and sugar content. The total soluble solids of the juice filtered using sterilized cotton cloth mesh was evaluated using a handheld refractometer (RF.5532 Euromex Brix hand refractometer, Euromex Microscopen bv, Arnhem, The Netherland). pH was determined with a pH meter (model PH–016, Kelilong Electron Co. Ltd, Beijing, China) calibrated with commercial buffers, pH 4 and 7. Titratable acidity was assessed by titrating 10 mL of diluted juice (1:5 juice-to-water, by volume) with 0.1 M NaOH using bromothymol blue solution as the visual endpoint indicator. The total acidity was calculated (as tartaric acid) by applying the standard method of OIV using the equation: total acidity (g/L) = 0.75 × volume of NaOH tartaric acid equivalents. A digital burette (Titrette, Brand Gmbh + Co KG, Wertheim, Germany) was used to determine the titration volume [15]. Sugar content of the filtered (using cellulose Whatman filter with pore size of 25 µm) 10 mL juice was analyzed by using the phenol-H_2_SO_4_ method following the procedure used by Nielsen [16]. Absorbance was measured at 490 nm using a spectrophotometer (UV-5100 Spectrophotometer, Metash Instruments Co. Ltd, Shanghai, China). The concentration of the sugar content was calculated using the calibration equation: sugar content = 0.0134x + 0.154, at R^2^ = 0.995, where x is concentration of dextrose glucose (mg/L). To determine antioxidant content of the juice sample, the juice was prepared as above for sugar analysis, and the phosphomolybdate method was applied following procedure used by Saeed et al. (2012). Ascorbic acid was used to develop the calibration equation: antioxidant content (mg/L AAE) = 0.0027x − 0.073, at R^2^ = 0.997, where x is concentration of ascorbic acid and AAE is ascorbic acid equivalent. 

The cactus pear juice pulp used as the fermentation substrate was adjusted to a sugar content of 210 g/L (expressed by dextrose) using table sugar. Tartaric acid was used as the acidulant during the pH adjustment. To inhibit bacteria growth, 70 mg/L sodium thiosulfate was added to 7 L of filtered juice. The must was preserved at 4 °C until fermentation started.

### 2.4. Fermentation

#### 2.4.1. Inoculum Preparation

The yeast cells (*S. cerevisiae* strain Montrachet, UCD 522) were incubated by adding 0.5 g of the dry yeast cells (hydrated in 50 mL mild hot distilled water at 35 °C) to a 150 mL conical flask containing 50 mL of sterilized YEPD media (1% *m/v* yeast extract, 2% *m/v* peptone, 2% *m/v* glucose), and the volume was increased to 150 mL using distilled water. The yeast cells were grown by incubating in a rotary shaker (VRN-200, Gemmy Industrial Corp., Taipei, Taiwan) with a speed of 120 rpm at 28 °C for 24 h. The yeast cells were then transferred into a 1000 mL Erlenmeyer flask, which contained 500 mL of the sterilized cactus pear juice pulp that had been previously adjusted to a pH of 3.4 using a pH meter (model PH-016, Kelilong Electron Co. Ltd, Beijing, China). The mixture was incubated at 28 °C for 36 h at a shaker speed of 150 rpm to use for the wine fermentation inoculation using the standard procedure described by OIV (2017) [17]. The number of viable yeast cells present, which was determined using the plate-count method, was 2 × 10^6^ CFU/mL in biomass. The inoculum concentration in Table 1 is the percentage suspension of 2 × 10^6^ CFU/mL (with a final magnitude order of 1, 1.6, 2.4, 3.2, and 3.8 × 10^5^ CFU/mL).

#### 2.4.2. Fermentation and Stabilization 

Inoculum concentration (2 × 10^6^ CFU/mL), pH, and fermentation temperature were adjusted based on the experimental design developed, as shown in Table 1. Two batches of the harvested fruit juice substrate were used to investigate the optimal fermentation parameters in the produced wine. The fermentation process was carried out in spherical 500 mL flasks containing 250 mL cactus pear juice. The fermentation mixture was mixed twice a day for 6 days. Plastic valve air locks were used on stationary fermenters for proper venting until the completion of fermentation (6 days). Total residual sugar was analyzed after 3 subsequent days using the phenol-H_2_SO_4_ method as per the procedure used by Nielsen [16]. A calibration equation was developed using dextrose glucose standard solution (5, 10, 20, 40, 60, and 80 mg/L). The concentration of total residual sugar was calculated using the calibration equation: residual sugar = 0.012x + 0.034, at R^2^ = 0.999, where *x* is concentration of dextrose glucose (mg/L). To set the center point of the experimental design and the end of fermentation, a trial fermentation was conducted at a temperature, pH, and inoculum concentration of 25 °C, 4, and 12%, respectively, based on the result reported by Peng et al. [11]. Tartaric acid solution (10 g/L) and 2 M sodium carbonate were used throughout the experimental design to adjust the required pH. During the 6th day, the fermentation settled and solid precipitate was formed. The total residual sugar content was 11 ± 0.03 g/L dextrose glucose equivalents.

Sulfur dioxide was used for biological stabilization and preventing oxidation of the final wine. To prevent potential sources of spoilage, the filtered wine was preserved by adding 50 mg/L of SO_2_. The settled (for 10 days at 4 °C) prickly pear young wine was filtered using sterilized cotton cloth mesh to remove the salt-cake. Subsequently, the wine was packaged in sterilized 330 mL brown bottles. Finally, the wine samples were used for analysis at room temperature.

### 2.5. Physicochemical Analysis of the Wine 

#### 2.5.1. Experimental Uncertainty

The sources of errors and uncertainties in the experiments from the measurements, processing conditions, and calibrations were considered in the analyses. Calibration equations of the spectroscopic method with R^2^ ≥ 0.99 was used, which assured less than 1% variation with the changes in x and y during the spectroscopic measurement and methods of standard solution preparation. The analysis results are expressed as the average of at least three measurements of the standard deviation to estimate the minimum variance point of the measured data. Before determining the standard deviation, outlying data were removed, and further analyses were carried out to fill the measurement gaps.

Batches of wine were produced at the same time with the same process conditions to identify the variation. Typically, the uncertainty of the replicated measured values of total acidity, alcoholic content, and total antioxidant content of the final wine were determined to be ±0.49 (g/L TTA), ±0.56 (%, *v/v*), and ±11.60 (mg/L AA), respectively. To determine the content of residual sugar, alcoholic, and total antioxidant levels in the juice substrate and the produced wine, an uncertainty calibration equation was used (Equation (1)) and *t*-statistics at a 95% confidence interval was applied [18].

(1) $$\;U_{x}=\;\frac{SE_{Yx}}{m}x\;\sqrt{\frac{1}{k}+\frac{1}{n}+\frac{{\left(Y_{x}-Y_{avg}\right)}^{2}}{m^{2}x{\left(n-1\right)}^{2}xS_{x}^{2}}}\;$$

where *U_x_*, *SE_Yx_*, *Y_x_*, *Y_avg_*, *k*, *m*, *n*, and *S_x_* stand for the standard uncertainty of the concentration of the analyte measured, standard error regression of the linear equation, average values of the replicate measurement, average signal for the calibration standards, number of replicates used to establish the sample’s average signal, slope, number of calibration standards, and variance of the calibration standard, respectively. 

#### 2.5.2. Total Acidity (TA)

The total acidity was estimated using the standard method described in OIV [15]. A digital burette (Titrette, Brand Gmbh + Co KG, Wertheim, Germany) was used to determine the titration volume. A 50 mL wine sample was vacuumed using a vacuum pump (VALUE, MR 1684, VACUUBRAND GMBH + CO KG, Wertheim, Germany) with continuous shaking for 2–3 min to reduce the carbon dioxide in the wine samples. From this, 10 mL was transferred into a 250 mL conical flask and mixed with 30 mL of distilled water (at 60 °C). For the endpoint color indicator, 1 mL bromothymol blue solution was added, and the solution was mixed well. Finally, it was titrated against standardized 0.1 N NaOH to the blue-green color endpoint. The endpoint color was compared with the reference color prepared by mixing 5 mL of commercial pH 7 buffer solution, 30 mL of distilled water (at 60 °C), 10 mL of decarbonated wine sample, and 1 mL of bromothymol blue solution in a cleaned 250 mL conical flask. The total acidity was calculated using the equation: total acidity *(*g/L*)* = 0.75 × volume of NaOH tartaric acid equivalents.

#### 2.5.3. Total Antioxidant Content

The total antioxidant content of the wine was determined using the phosphomolybdate method following the procedure used by Saeed et al. with some modifications. Ascorbic acid (7.5 × 10^3^ mg L^−1^) was diluted to prepare series of standard solutions (50, 100, 150, 200, 250 mg L^−1^ using distilled water). A blank solution was prepared by mixing 0.3 mL of distilled water and 3 mL of phosphomolybdate reagent (0.6 M sulfuric acid, 28 mM sodium phosphate, and 4 mM ammonium molybdate). Three milliliters of phosphomolybdate reagent solution was added to 0.3 mL of the standard solution at room temperature. A volume of 0.3 mL of the wine sample was prepared using a method similar to the standard solution. Finally, all standards, samples, and blank were incubated in a water bath at 95 °C for 90 min using aluminum foil-capped test tubes. After the solutions were sufficiently cooled to room temperature, absorbance was measured at 765 nm against the blank using a spectrophotometer (UV-5100 Spectrophotometer, Metash Instruments Co. Ltd, Shanghai, China). The ascorbic acid content (mg/L AAE) was calculated using the calibration equation: absorbance reading = 0.003x − 0.029, at R^2^ = 0.990, where x is concentration of ascorbic acid (mg/L AAE).

#### 2.5.4. Estimating Alcohol Content 

Spectrometric determination of ethanol (%, *v/v*) using acidified dichromate solution by microdistillation of the samples, which is routinely used in many winery laboratories, was adopted in this study [19]. Using the procedure applied by Babu et al.: into 500 mL fractional distillation flask, 1 mL of wine sample and 30 mL distilled water were placed [20]. During the distillation, 20 mL of the distillate was collected in a 50 mL receiving flask that contained 25 mL of potassium dichromate solution (0.17 M K_2_Cr_2_O_7_ dissolved in 5.9 M H_2_SO_4_). The standard and sample were incubated at 62.5 °C in a water bath for 20 min. After cooling to room temperature, the volume was increased to 50 mL using distilled water. Finally, the absorbance of the solutions was measured at 600 nm using a spectrophotometer (UV-5100 Spectrophotometer, Metash Instruments Co. Ltd, Shanghai, China) against the blank solution containing 25 mL reagent and 30 mL distilled water. The standard curve was developed using 2, 4, 8, 12, and 16 (%, *v/v*) standard ethanol solution in distilled water that was prepared similarly to sample solutions. The wine sample ethanol content was estimated by calculating using the calibration equation: absorbance reading = 0.037x − 0.008, at R^2^ = 0.9992, where x is concentration of ethanol (%, *v/v*). 

#### 2.5.5. Sensory Evaluation 

Five females and five males in the age group of 24–45 years were trained to evaluate the sensory quality of the wine samples. Accordingly, they were allowed to evaluate the overall sensorial acceptability of the produced wine. The evaluation score was based on 9-point hedonic scale that included color, aroma, and taste. The evaluation method was based on the procedure used by Wichchukit et al. [21]. The method uses human behavior as its data source, rather than numerical estimates obtained from the ratings. Moreover, the 9-point hedonic scale encourages judges to re-taste if they have forgotten their response to the stimulus; such double-checking minimizes error sources. Similar-sized and -colored wine glasses were filled to 1/3 capacity with coded and randomly ordered wine samples (at 15 °C) and covered with watch glasses. Each panelist evaluated two wine samples, including the replicate samples, based on the developed experimental design. The evaluation was performed in well-ventilated individual booths prepared as a sensory analysis room at 20 °C. Rinsing between samples was done with mineral water. The scores were used to evaluate the overall quality of the wine obtained under optimized conditions.

### 2.6. Experimental Design and Data Analysis 

Experimental design and data generation were performed by response surface analysis coupled with central composite rotatable design (CCRD) using Design-Expert version 10.0.3 (Stat-Ease Inc., Minneapolis, MN, USA). The CCRD experimental design with both coded and actual values are shown in Table 1. Moreover, Table 1 confirms the domain and range of the minimum and maximum limits of the three experimental variables studied for the optimizing process. The CCRD with a quadratic model was used to investigate the overall effect of independent variables, such as fermentation temperature, pH, and inoculum concentration. The CCRD included 20 experiments formed by 6 central points and 6 (α = ±1.682) axial points for a 2^3^ full factorial design was used (Table 2).

Experimental results were measured three times and expressed as mean ± standard deviation to the nearest two-digit values. Calibration curves (standard curves) were used to determine the quantity of sample during spectroscopic measurements. To identify the optimized fermentation parameters, the measured experimental data were fitted with multiple linear regression techniques to create a second-order surface response regression equation, as shown below:

(2) $$\;Y=\;\beta_{0\;}+\sum_{i=1}^{3}\beta_{i}x_{i}\;+\;\sum_{i=1}^{3}\beta_{ii}x_{i}^{2}+\;\sum_{i=1}^{3}\sum_{j=\left(i+1\right)}^{3}\beta_{ij}x_{i}x_{j}\;$$

where *Y* stands for those responses to be predicted (alcohol content, antioxidant content, titratable acidity, and sensory score value of cactus pear fruit wine); *i*, *j* represent linear and quadratic coefficients, correspondingly; $x_{i}$ and $x_{j}$ correspond to the three independent variables: fermentation temperature, pH, and inoculum concentration; *β*_0_ (intercept), *β_i_* (linear effects), *β_ii_* (squared effects), and *β_ij_* (interaction terms) were used for regression coefficients.

## 3. Results and Discussion

### 3.1. Statistical Analysis

The final quality-affecting parameters of cactus pear fruit juice fermentation (fermented for 6 days and further matured for 10 days) are shown in Table 2. The fermentation substrate was inoculated with *S. cerevisiae* strain Montrachet, UCD 522, developed using cactus pear fruit juice biomass. These empirical values were applied in a multiple regression analysis using response surface analysis to fit the second-order polynomial equations. This quadratic function is one of the successful applications of RSM that practically approximates many fermentation systems [22]. Analysis of variance (ANOVA) was conducted to investigate the statistical significance of the models, and *p-*values were less than 0.005. Coefficients of determination (R^2^), adjusted R^2^ values, standard deviations, and adequacy precision of the four dependent variables were determined (presented in Table 3) to validate the developed model’s goodness of fit. Coefficients of the developed models were significantly fitted (*p* < 0.05) to the response data.

### 3.2. Effects of Independent Variables on the Final Wine

Agroclimatic conditions of the site (Adigrat, Northern Ethiopia) at which samples were collected are semi-dry from January to March (about 28 °C), but April–September (about 21 °C) is the wet season. It is well documented that the best fruit-picking time for obtaining high-quality physicochemical characters of cactus pear is in March and April [23]. Total acidity (g/L tartaric acid equivalents), sugar content (g/L dextrose equivalents), antioxidant content (mg/L AAE), degree Brix, and pH of the fruit juice during preliminary characterization was measured as 1.8 ± 0.37, 130 ± 0.02, 179.4 ± 5.48, 17 ± 0.23, and 3.9 ± 0.61, respectively. The degree of ripeness, fruit variety, origin, and harvesting period affects the initial sugar content, total acidity, and pH of cactus pear fruit juice composition [24]. 

An efficient fermentation process is expected to convert approximately 17 g/L of sugar into 1%, *v/v* alcohol. This can be achieved if the fermentation must’s (yeast) nitrogen level is adjusted and pectinase (for homogenization) is used [10]. In the current study, adjustment of the must’s nitrogen level and the addition of a homogenizer was not considered. Due to these, the current optimum fermentation process was expected to produce about 12%, *v/v* alcohol. However, the volatilization of alcohol during filtration, and unadjusted yeast nitrogen level lead to decrease the content of alcohol to about 9%, *v/v*.

Regression models (Equations (2)–(5)) were utilized to express the relationship of temperature, pH, and inoculum concentration with total acidity, alcohol content, total antioxidant content, and sensory acceptance of the produced wine. Regression coefficients of the developed quadratic polynomial models showed significant (*p* < 0.05) relationships between the independent variables and total acidity, alcohol content, antioxidant content, and sensory acceptance of the final wine. The final reduced model equations with a significant coefficient of parameters (*p* < 0.05) were used to predict data and are shown in Equations (2)–(5).

The models in Equations (2)–(5) produced *F*-values of 6.10, 18.36, 14.59, and 8.77, respectively. These values indicate that there is a significant (*p* < 0.005) relationship between independent and dependent variables, thus, they can be used to predict quality-related responses of the wine. There was only a 0.5% probability that noise led to these models’ *F*-values being large enough to adequately predict the total acidity, alcohol content, total antioxidant content, and sensory acceptance of the cactus pear juice wine.

(3) $$\begin{array}{l} TA\;\left(g/L\;tartaric\;acid\right)=-86.63+1.26\times Temp.+38.47\times pH+0.17\times IC\\ -0.29\times Temp.\times pH-3.78\times pH^{2} \end{array}\;$$

(4) $$\begin{array}{l} \begin{array}{l} Alc \end{array}\left(\%v/v\right)=-8.74-0.09\times Temp.+7.29\times pH+0.01\times IC\\ +0.02\times Temp.\times IC-0.83\times pH^{2}-0.01\times IC^{2} \end{array}\;$$

(5) $$\begin{array}{l} Anc\;\left(mg\;AAE/L\right)=-456.23+0.95\times Temp.+275.06\times pH+4.54\times IC\\ -2.07\times Temp.\times pH+0.26\times Temp.\times IC+0.13\times Temp.^{2}-26.51\times pH^{2}-0.31\times IC^{2} \end{array}\;$$

(6) $$\begin{array}{l} Sensory=8.26+0.36\times Temp.-2.64\times pH+0.15\times IC\\ +0.08\times Temp.\times pH+0.01\times Temp.\times IC-0.02\times Temp.^{2}-0.01\times IC^{2} \end{array}\;$$

where *Temp*, *IC*, *Alc*, *TA*, and *Anc*, stand for temperature, inoculum concentration, alcohol content, total acidity, and antioxidant content, correspondingly. These model equations are valid only for the *S. cerevisiae s*train Montrachet, UCD 522 developed from cactus pear fruit biomass. 

### 3.3. Optimization Process Parameters of the Fermentation 

The fermentation process was optimized for temperature, pH, and inoculum concentration (IC) for predicting total acidity, alcoholic content, total antioxidant content, and sensory quality of the final wine. The optimized process parameters were applied within the selected ranges of temperature, pH, and IC of 16–36 °C, 3.1–4.7, and 5–19 (%, *v/v*), respectively. For the desirability function, 0.603 was selected as the optimum value. The six readings of the optimum values obtained at this desirability level were close to each other; temperature, pH, and IC varied within ranges of 29.710–29.733 °C, 3.896–3.910, and 16–16.001(%, *v/v*), respectively. From the ANOVA of the response surface quadratic model, there was a significant effect (*p* < 0.05) of the independent fermentation variables on the total acidity of the wine, producing first-order linear effects (Temp, pH, and IC) and a second-order quadratic effect for pH (pH^2^). However, the interactive effects (Temp × pH, Temp × IC, pH × IC) on the second-order quadratic effect (Temp^2^) were significant at *p* < 0.1. On the other hand, the second-order quadratic effect (IC^2^) had an insignificant effect (*p* > 0.1) on the total acidity of the wine produced. This is because of the inoculum strain, which depends on the type of acid present, temperature, and pH conditions for facilitating the production of total acids. At higher temperature and lower pH, the concentration of yeast could decrease. According to a study by Torija et al. viable cells decreased at high temperatures, especially at 35 °C [25]. A decrease in the concentration of yeast cells slows the fermentation process, which, in turn, suppresses the extraction of total acids. 

**Figure 1:**
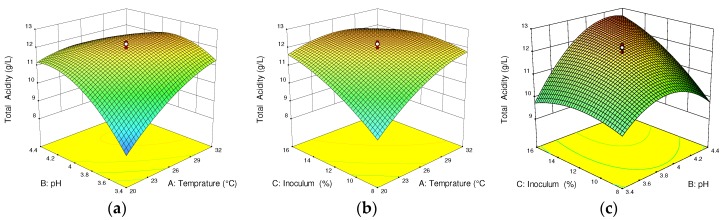
Response surface and contour plots for the total acidity of cactus wine: (**a**) interactive effect of pH and temperature; (**b**) inoculum concentration and temperature; (**c**) inoculum concentration and pH, respectively. In each plot, the third fermentation parameter is fixed at the center point value.

The quadratic response 3D surface plot in Figure 1 illustrates the optimization of fermentation temperature, pH, and inoculum concentration for total acidity of the produced wine. The dominant acid present in cactus pear fruit is citric acid (62 mg/100 g) [26]. In the current study, the acidulant used for pH adjustment was tartaric acid, which is related to the final flavor of the wine, as well as its ability to increase pH during pH adjustment. Total acidity can be varied from 7.5 to 8.6 g/L tartaric acid equivalents if *S. cerevisiae* strains AS2 and AS4 are used [27]. Moreover, Phutela and Kaur [28] reported that total acid varied in the range of 0.62–0.74% when the inoculum size increased from 5 to 10% using the *S. cerevisiae* NRRL Y–2034 strain. In the current study, *S. cerevisiae* strain Montrachet, UCD 522 was used. The metabolism of total acidity tartaric acid equivalents of the final wine is not significant due to a combination of differences in ethanol concentration and corresponding potassium tartrate precipitation and some bacterial growth, which is similar to the study by Reynolds et al. [29]. 

As clearly shown in Figure 1a, at a constant inoculum concentration (12%), the total acidity gradually increased to 11.9 g/L Tartaric acid equivalent (TTAE) at pH and temperature of 3.9 and 29 °C, respectively. The increased total acidity is due to the tartaric acid added during pH adjustment, which may have inhibited some bacterial growth. Further, Figure 1b shows that the effect of inoculum concentration and temperature insignificantly increased the total acidity to 12 g/L. Conversely, Figure 1c shows that the total acidity of the wine was slightly increased to 12.3 g/L due to a significant interaction effect of pH at a constant temperature of 26 °C. In Figure 1c, the larger pH resulting in increased total acidity illustrates a linear interaction between pH and total acidity during the fermentation. This is due to the fact that tartaric acid was used as the acidulant, which favored the increase in total acidity. Generally, the model equation of total acidity had a maximum response of 11.53 ± 1.32 g/L TTAE at a temperature of 26 °C, pH of 3.9, and inoculum concentration of 12%. 

Temperature, pH, and inoculum concentration are prominent variables for determining the alcoholic content and quality of a wine [14]. From the ANOVA of the response surface quadratic model, all three independent variables influenced (*p* < 0.05) the alcoholic content of the wine. Similarly, the interaction effect of temperature and inoculum concentration (Temp × IC) had a significant effect (*p* < 0.05) on the wine’s alcoholic content. Furthermore, the quadratic effects of the inoculum concentration and pH were significant at *p* < 0.1. However, the interaction effect of temperature and pH, as well as inoculum concentration and pH, have insignificant effects (*p* > 0.1) on the alcoholic content of the wine.

**Figure 2:**
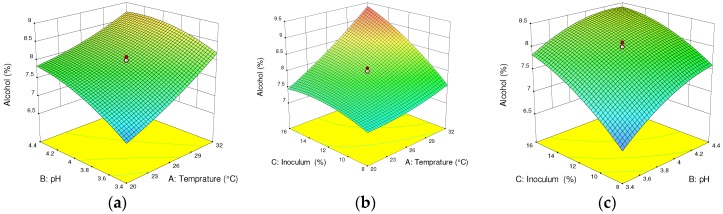
Response surface and contour plots for alcohol content of cactus wine. (**a**) Interactive effect of pH and temperature; (**b**) inoculum concentration and temperature; (**c**) inoculum concentration and pH. In each plot, the third fermentation parameter is fixed at the center point value.

As shown in Figure 2a, the alcohol content increased to a greater degree in response to temperature compared to pH, which had less of an effect, at constant inoculum concentration of 12%. The optimum temperature and pH to produce 7.9%, *v/v* alcohol content were 26 °C and 3.9, correspondingly. This clearly shows that the effect of temperature on the fermentation rate is positive due to the fact that a high alcohol content was produced with the facilitated fermentation process. Figure 2b shows that alcohol content during the fermentation process increased progressively with increasing inoculum concentration and temperature. The optimum temperature and inoculum concentration at a constant pH of 3.9 to produce 8.1 ± 0.313 (%, *v/v*) alcohol are 26 °C and 12%, respectively. Further increment of temperature can suppress the growth of yeast cells, as inoculum concentration has shown to have an insignificant effect from 14% to 16%. Babu et al. reported that increasing the temperature beyond 30 °C decreased alcohol content from 9.2 to 6.8% [20]. Also, due to the consumption of substrates in the fermentation process, further production of alcohol was inhibited, which might be attributable to the decline in yeast fermentation activity and population. In general, the overall optimum temperature, pH, and inoculum concentration to produce alcohol content of 9.4 ± 0.313 (%, *v/v*) were 26 °C, 3.9, and 12%, correspondingly.

From the ANOVA of the response surface quadratic model, all the linear coefficients (independent parameters) and the quadratic effect of the inoculum concentration show significant effects (*p* < 0.05) on the antioxidant content of the produced wine. Furthermore, the interaction effects of temperature with pH and temperature with inoculum concentration (Temp × pH; Temp × IC), the quadratic effect of temperature, as well as the inoculum concentration had significant effects on the antioxidant content at *p* < 0.1. However, the interaction effect of inoculum concentration and pH (pH × IC) had no significant effect (*p* > 0.1). 

Investigation of the influence of temperature, pH, and inoculum concentration on the total antioxidant content of the final wine clearly shows that there is a positive influence of temperature and inoculum concentration. Moreover, their interaction had a significant effect on antioxidants production during the fermentation process, as compared to the effect of pH. This is consistent with the study of Ivanova et al. (2012) [30] on improving the polyphenol concentration of wine products at a controlled temperature, pH, inoculum concentration, and a specified addition of SO_2_.

**Figure 3:**
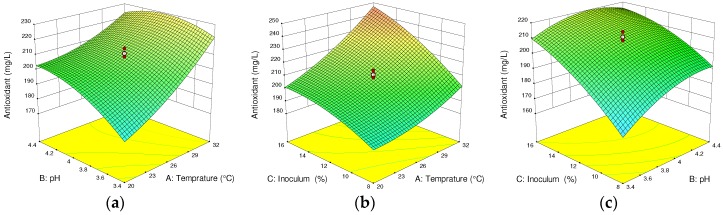
Response surface and contour plots for antioxidant content of cactus wine. (**a**) Interactive effect of pH and temperature; (**b**) inoculum concentration and temperature; (**c**) inoculum concentration and pH. In each plot, the third fermentation parameter is fixed at the center point value.

From the 3D graph in Figure 3a of the quadratic response surface plotted for identifying the maximum total antioxidant, temperature (29 °C), and pH indicates that the fermentation process was influenced to increase total antioxidants to 211 mg/L. This phenomenon is expected, since the maximum temperature enhances the production of total antioxidants due to the facilitated rate of the fermentation process (Jacobson, 2006) [10]. The optimum fermentation temperature and pH at a constant inoculum concentration (12%) to produce 211 mg/L total antioxidants is 26 °C and 3.9, respectively. However, as shown in Figure 3b, the effect of inoculum concentration and temperature lowered the total antioxidant content to 205.8 mg/L. The optimum temperature and inoculum concentration with a constant pH (3.9) to produce 205.8 mg/L was 26 °C and 12%, respectively. This indicates that the interactive influence of temperature and inoculum concentration on total antioxidant capacity of the produced wine is less than the interactive influence of temperature and pH. This could be due to the higher temperature (>30 °C), resulting in a decrease in the population of yeast cell activities.

Increasing the inoculum concentration (to about 12%) and decreasing pH (to about 4.2) at constant temperature (26 °C) had a substantial influence on the total antioxidant concentration, which is 211 mg/L, as shown in Figure 3c. The main reason for the increasing and decreasing of the total antioxidant content during cactus pear fruit fermentation could be due to the compounds present in the fruit, and to the synergistic effects of the interaction among certain metabolic products formed during the fermentation process. This phenomenon is consistent with the study by Ayed and Moktar [7]. In their study, the optimum temperature, pH, and inoculum concentration to produce a total antioxidant concentration of 208.35 ± 9.15 (mg/L) AAE were recorded as 26 °C, 3.9, and 12%.

**Figure 4:**
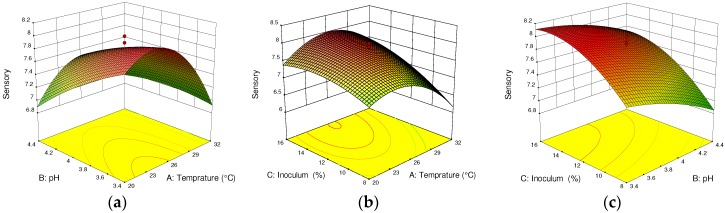
Response surface and contour plots for sensory acceptance of cactus wine. (**a**) Interactive effect of pH and temperature; (**b**) inoculum concentration and temperature; (**c**) inoculum concentration and pH. In each plot, the third fermentation parameter is fixed at the center point value.

As can be seen from Figure 4a, the overall sensory acceptance of the wine increased to 7.9 when the temperature during the fermentation process was 26 °C, the pH value was 3.9, and the inoculum concentration was constant (12%). However, their interaction effect was less significant (*p >* 0.05). The 3D graph shown in Figure 4b illustrates this; the interaction of the inoculum concentration (12%) and temperature (26 °C) was less significant, producing an overall sensory acceptance of 7.9. Figure 4c shows that there are insignificant (*p >* 0.1) interaction effects of inoculum concentration and pH, even though the sensorial acceptance was increased as the inoculum concentration increased. This could be due to the yeast inoculum concentration suppressed by the development of other microbial contaminants at lower pH (4.4). From the ANOVA of the response surface quadratic model for sensory acceptance, the cactus wine fermentation process was affected significantly (*p* < 0.05) and linearly by temperature, pH, and inoculum concentration. Additionally, quadratic effects of temperature (Temp^2^) and inoculum concentration (IC^2^) showed significant effects (*p* < 0.05) for overall sensorial acceptance of the final wine. The interaction effects of temperature with pH (Temp × pH) and temperature with inoculum concentration (Temp × IC) significantly affected the overall sensory acceptance at *p* < 0.1. The linear effect of temperature, pH, and inoculum concentration, as well as the interaction effect of temperature with inoculum concentration (Temp × IC), is consistent with the report by Peng et al. [11], who studied the apple wine fermentation process. The optimum fermentation parameters to produce a 7.74 ± 0.34 overall sensorial acceptance value were a temperature of 26 °C, pH of 3.9, and inoculum concentration of 12%.

### 3.4. Validating Output Parameters of the Predictive Model

The predictive models of the final wine response variables were validated based on R-square, adjusted R-square, and coefficient of variance. As shown in Table 3, the coefficients of determination—R-square, adjusted R-square, and coefficient of variance—indicate that the examined factors had significant effects on the alcohol content, total antioxidant content, and sensory acceptance. The relationship among independent factors versus alcohol content, total antioxidant content, and sensory acceptance of the final wine were real and reliable. The low value of the coefficient of variance (<5%) for alcohol content, total antioxidant content, and sensory acceptance indicates good precision and reliability of the models. R^2^ > 0.85 for models of alcohol content, total antioxidant content, and sensory acceptance indicates that the regression explained the relationships well. However, the R^2^ < 0.85 of the model for total acidity is less significant, and 37% of the total variation is not explained by the model. The difference of the adjusted R-square and predicted R-square, as shown in Table 3 for alcohol content, total antioxidant content, and sensory acceptance, respectively, is approximately 0.20, supporting the significance of the models. These regression models were used to predict the response factors of the wine in the experiments for the purpose of model adequacy verification. Moreover, adequacy precisions of 7.27, 16.50, 14.32, and 11.34 show that the models had an adequate signal to predict total acidity, alcohol content, total antioxidant content, and sensory acceptance of the wine produced, respectively, since an adequacy of precession greater than 4 is desirable. However, the validity of the optimal combination of predicted fermentation parameters was tested by confirmatory experiments under the overall predicted combination input variables, which is shown in Table 4.

### 3.5. Simultaneous Optimization of the Fermentation Parameters for Overall Responses of the Wine

To generate the desired response values, total antioxidant content (AAE, mg/L) and sensorial acceptance were aimed to be maximized, as it is required to increase their values. However, the alcohol content (%, *v/v*) of a commercial wine should be in the range of 8–15%, thus, the study aimed to produce an alcohol % within that range [31]. The total acidity was targeted at 13.1 g/L of tartaric acid as of the standard limit of OIV [32]. The predicted alcoholic content of the final wine was in the range 8.98 ± 0.31%, *v/v*, and this value is similar to the alcohol content (9.93%, *v/v*) analyzed by Navarrete-Bolaños et al. in Mexican cactus pear fruit [3]. The total antioxidant content (235.3 ± 9.15 mg/L AAE) of the predicted value is similar (230.0 mg/L AAE) to the value reported by Kelebek et al. [33]. The presence of ascorbic acid in wine is an indicator of antioxidant content. However, the predicted total antioxidant content is less than the value (427.81 mg/L AAE) determined by Owusu et al. in tomato wine, which could be due to the difference in the nature of the fruits and the fermentation method used [34]. The expected value of sensorial acceptance (7.74 ± 0.34) of the wine is comparable to the sensory analysis (6.16 ± 0.31) reported by De Castilhos et al. in Bordo grape wine produced by submerged static pomace wine making processes [35]. The predicted total acidity of 12.39 ± 1.32 g/L tartaric acid equivalents is comparable to the value (13.4 g/L tartaric acid equivalents) determined by Ray et al. in purple sweet potato wine [36]. 

### 3.6. Validating the Fermentation Parameters’ Combined Effects on Desired Responses of the Wine

The desirability 0.6 was generated by Design-Expert version 10.0.3 to set the overall optimum values during simultaneous optimization. Four responses were assigned: low (8%, *v/v*) and high (15%, *v/v*) alcohol content values, total acidity in the range 13.1 g/L TAE, as well as maximized total antioxidant content and sensory acceptance. To achieve simultaneous optimization of multiple responses, the least desirable value is considered zero and the most desirable is one [37].

To see the predicted optimum antioxidant content, acceptable alcoholic content, total acidity, and sensorial acceptance, a confirmatory experiment was carried out by fermenting the cactus pear fruit juice at a temperature of 30 °C, pH value of 3.9, and yeast inoculum concentration of 16%. The validity of the expected optimum combination of the fermentation process parameters were compared with the confirmatory experimental values. Table 4 shows the predicted total acidity of 12.4 g/L ± 1.32 tartaric acid equivalents, alcohol content of 9 ± 0.31 (%, *v/v*), total antioxidant content of (235 ± 9.15) mg/L AAE, and sensory acceptance of 7.74 ± 0.34. The predicted optimal response parameters are comparable at a 95% confidence level to the confirmatory experimental values of total acidity 12.26 ± 0.49 g/L tartaric acid equivalents, alcoholic strength of 8.89 ± 0.56 (%, *v/v*), total antioxidant concentration of 221 ± 11.6 mg/L AAE, and sensory acceptance of 8.1 ± 0.72. The residual sugar content was 5.32 ± 0.02 g/L dextrose equivalents in the produced dry wine. This indicates that the optimized fermentation process produced wine with a residual sugar of 5.32 ± 0.02 g/L dextrose equivalents.

As shown in Table 4, the measured data have fallen within range of the predicted interval (95% PI low and high), which indicates that response values perfectly matched the predicted response values of the produced wine. The small interval between the two experimental runs indicates good precision in the estimate. The optimum fermentation temperature recorded is in agreement with the study by Arrizon et al. on the cactus distilled beverage fermentation process (30–33 °C) [24]. Even though the optimized pH value is greater than the pH value (3.6) to produce a quality wine from apple [11], it is in a comparable range to the pH value (3.2–4.0) needed for the grape fermentation process to get a quality wine, as explained by Jacobson [10]. The optimum inoculum concentration is larger than the optimum value (9%) determined by Peng et al. [11], which may be due to the difference in the fruits’ physicochemical composition. 

### 3.7. Limitation of the Study

The cactus pear fruit juices were homogenized manually to carry out this study. Instead of manual homogenization, using enzymes may be key to a better extraction of aromatic compounds, enhancing the wine quality. However, the selection of proper enzymes needs careful attention to avoid undesirable wine quality. Furthermore, this study was performed using a single yeast strain due to the scope of the work. These results can be used as a baseline for further investigating the effects with two or more yeast strains for this fruit’s wine production, which may address the limitation.

## 4. Conclusions

This work was aimed at predicting the optimal fermentation conditions for wine production from cactus pear fruit. The developed predictive models for all responses of interest of the wine yielded results that are predictable, controllable, and reproducible, with predicted results closely agreeing (95%) with the experimental values determined during the confirmatory experiment. Regression coefficients of the developed quadratic polynomial models showed significant (*p* < 0.05) relationships within the dependent variables, respective to quality-indicative responses of the final wine. Maximum total acidity (12.39 ± 1.32 g/L tartaric acid equivalents), alcohol content (9 ± 0.31%, *v/v*), total antioxidant (235.3 ± 9.15 mg/L AAE), and sensory acceptance (7.74 ± 0.34) of the cactus wine were obtained at an optimized temperature of 30 °C, pH of 3.9, and yeast inoculum concentration of 16%.

## Figures and Tables

**Table 1:** The CCRD experimental design of experimental runs and their coded values.

Factors	Factor Levels				
	−α	−1	0	1	+α
Temperature (°C)	16	20	26	32	36
pH	3.1	3.4	3.9	4.4	4.7
Inoculum concentration (%, *v/v*)	5	8	12	16	19

CCRD: central composite rotatable design.

**Table 2:** Three-factor CCRD experimental design of all experimental runs with their coded values and measured values for the dependent variables.

Std	Run	Temp. (°C)	pH	IC (%)	Y_1_	Y_2_	Y_3_	Y_4_
					TA (g/L Tartaric Acid)	Alc (%, *v/v*)	Anc (mg/L AAE)	Sensory
**1**	20	(−1)20	(−1)3.4	(−1)8	8.2	6.2	147.3	7.6
**2**	10	(+1)32	(−1)3.4	(−1)8	11.9	7.3	190.5	6.3
**3**	14	(−1)20	(+1)4.4	(−1)8	9.4	7.9	189.8	6.1
**4**	5	(+1)32	(+1)4.4	(−1)8	10.9	7.4	187.6	5.8
**5**	11	(−1)20	(−1)3.4	(+1)16	7.6	7.2	190.8	8
**6**	6	(+1)32	(−1)3.4	(+1)16	9.8	8.8	238.6	7.7
**7**	3	(−1)20	(+1)4.4	(+1)16	13.1	7.4	195.8	6.7
**8**	2	(+1)32	(+1)4.4	(+1)16	10.5	9.4	239.3	7.4
**9**	12	(−1.682)16	(0)3.9	(0)12	8.5	7.1	198.7	6.7
**10**	8	(+1.682)36	(0)3.9	(0)12	11.8	9.4	254.7	5.6
**11**	1	(0)26	(−1.682)3.1	(0)12	7.8	6.5	178.9	7.5
**12**	19	(0)26	(+1.682)4.7	(0)12	9.7	8.3	210.6	7.7
**13**	9	(0)26	(0)3.9	(−1.682)5	7.9	6.5	178.5	6.8
**14**	4	(0)26	(0)3.9	(+1.682)19	13.1	8.4	220.9	7.6
**15**	18	(0)26	(0)3.9	(0)12	12	7.8	205.8	8
**16**	7	(0)26	(0)3.9	(0)12	11.6	7.9	201.3	7.9
**17**	13	(0)26	(0)3.9	(0)12	12.3	8.1	205.7	7.8
**18**	17	(0)26	(0)3.9	(0)12	12.2	8	211	7.7
**19**	16	(0)26	(0)3.9	(0)12	11.9	7.9	214.3	7.9
**20**	15	(0)26	(0)3.9	(0)12	11.8	8.1	208.7	7.7

Where Std, Run, Temp, IC, Alc, TA, AAE and Anc represent standard order, run order, temperature, inoculum concentration, alcohol content, total acidity, ascorbic acid equivalent and antioxidant content, respectively. Measured data are mean values of the three replicated trials.

**Table 3:** Experimental data analysis for all predictive responses models.

Statistical Parameters	Y_1_	Y_2_	Y_3_	Y_4_
	TA (g/L Tartaric Acid)	Alc (%, *v/v*)	Antc (mg AAE/L)	Sensory
Std. Dev.	1.32	0.31	9.15	0.36
Mean	10.60	7.78	203.44	7.22
C.V. %	12.42	4.03	4.50	4.69
PRESS	56.11	3.73	4543.25	4.92
R-Squared	0.6237	0.9109	0.9159	0.8778
Adjusted R-Squared	0.4894	0.8697	0.8548	0.8066
Predicted R-Squared	0.1296	0.7397	0.5853	0.5629
Adequacy of Precision	7.267	16.496	14.317	11.340

Where Std. Dev., C.V., TA, Alc, Antc, and PRESS stands for standard deviation, coefficient of vitiation, total acidity, alcoholic content, antioxidant content, and predicted regression error sum of squares, respectively.

**Table 4:** Confirmation report to validate the combination of fermentation parameters.

Two-sided Confidence = 95% *n* = 2								
Factor	Name	Optimum Level	Low Level	High Level	Coding			
A	Temp (°C)	29.71	20.00	32.00	Actual			
B	pH	3.90	3.40	4.40	Actual			
C	IC (%)	16.00	8.00	16.00	Actual			
**Response**	**Predicted** **Mean**	**Predicted** **Median**	**Std Dev**	***n***	**SE Pred**	**95% PI Low**	**Measured Data Mean**	**95% PI High**
TA (g/L TTA	12.3974	12.3974	1.31622	2	1.43	9.33	12.26	15.47
Alc (%, *v/v*)	8.98313	8.98313	0.313497	2	0.35	8.23	8.89	9.74
Anc (mg/L AAE)	235.304	235.304	9.14966	2	10.20	212.85	221.00	257.76
Sensory	7.74112	7.74112	0.338527	2	0.37	6.93	8.10	8.55

*n*, Temp, IC, TA, Alc, Anc, SE Pred, Std Dev, g/L TTA and PI represent number of confirmations, temperature, inoculum concentration, total acidity, alcoholic content, antioxidant content, standard error prediction, standard deviation, gram per litter tartaric acid and predicted interval, respectively.

## Supplementary Materials

The following are available online at http://www.mdpi.com/2304-8158/7/8/121/s1, Table S1: ANOVA for response surface quadratic model of total acidity, Table S2: ANOVA for response surface quadratic model of alcohol content, Table S3: ANOVA for response surface quadratic model of total antioxidant properties, Table S4: ANOVA for Response Surface Quadratic model of sensory quality of the wine.
