# Supplementary material for: Optimization of Cactus Pear Fruit Fermentation Process for Wine Production
Source: Foods. 2018 Jul 30;7(8):121. doi: 10.3390/foods7080121 (PMC6111886; doi:10.3390/foods7080121)
Supplement: Supplementary file 1 [file foods-07-00121-s001.pdf]

Supplementary Materials: Optimization of Cactus Pear Fruit Fermentation Process for Wine Production

Zenebe Tadesse Tsegay [1,2], Chanukya Basavanahally Sathyanarayana [2], Solomon Mengistu Lemma [2,3,*]

Supplemental Table (ANOVA)

Table S1. ANOVA for response surface quadratic model of total acidity.

Source of error	Sum of Squares	df	Mean Square	*F* Value	*p*-value or Prob > F	
Model	54.62807	9	6.069785	6.173541	0.004396	significant
A-Temp	7.843737	1	7.843737	7.977817	0.018021	
B-pH	6.741803	1	6.741803	6.857047	0.025659	
C-Inc.	6.394961	1	6.394961	6.504276	0.028847	
AB	6.125	1	6.125	6.2297	0.031667	
AC	3.92	1	3.92	3.987008	0.073787	
BC	4.5	1	4.5	4.576922	0.058096	
A^2	4.131345	1	4.131345	4.201966	0.067523	
B^2	15.30065	1	15.30065	15.5622	0.002753	
C^2	2.442384	1	2.442384	2.484134	0.146078	
Residual	9.831934	10	0.983193			
Lack of Fit	9.498601	5	1.89972			
Pure Error	0.333333	5	0.066667			
Core Total	64.46	19				

Table S2. ANOVA for response surface quadratic model of alcohol content.

Source	Sum of Squares	df	Mean Square	*F* Value	*p*-value Prob > F	
Model	13.51	9	1.50	18.36	<0.0001	significant
A-Temp	4.77	1	4.77	58.27	<0.0001	
B-pH	2.32	1	2.32	28.35	0.0003	
C-Inc.	3.79	1	3.79	46.35	<0.0001	
AB	0.18	1	0.18	2.20	0.1688	
AC	1.13	1	1.13	13.75	0.0041	
BC	0.12	1	0.12	1.53	0.2446	
A^2	0.15	1	0.15	1.89	0.1992	
B^2	0.56	1	0.56	6.83	0.0259	
C^2	0.46	1	0.46	5.66	0.0387	
Residual	0.82	10	0.082			
Lack of Fit	0.74	5	0.15			
Pure Error	0.073	5	0.015			
Core Total	14.33	19				

Table S3. ANOVA for response surface quadratic model of total antioxidant properties.

Source	Sum of Squares	df	Mean Square	*F* Value	*p*-value Prob > F	
Model	10170.40	9	1130.04	14.59	0.0001	significant

A-Temp	3755.87	1	3755.87	48.50	<0.0001
B-pH	712.06	1	712.06	9.19	0.0126
C-Inc.	3563.62	1	3563.62	46.02	<0.0001
AB	308.76	1	308.76	3.99	0.0738
AC	316.26	1	316.26	4.08	0.0709
BC	143.65	1	143.65	1.85	0.2031
A^2	314.10	1	314.10	4.06	0.0717
B^2	632.99	1	632.99	8.17	0.0170
C^2	342.83	1	342.83	4.43	0.0617
Residual	774.41	10	77.44		
Lack of Fit	670.45	5	134.09		
Pure Error	103.96	5	20.79		
Core Total	10944.81	19			

Table S4. ANOVA for Response Surface Quadratic model of sensory quality of the wine.

Source	Sum of Squares	df	Mean Square	F Value	*p*-value Prob > F	
Model	9.99	9	1.11	8.77	0.0011	Significant
A-Temp	0.68	1	0.68	5.38	0.0428	
B-pH	0.78	1	0.78	6.16	0.0324	
C-Inc.	2.09	1	2.09	16.53	0.0023	
AB	0.50	1	0.50	3.95	0.0750	
AC	0.50	1	0.50	3.95	0.0750	
BC	0.020	1	0.020	0.16	0.6994	
A^2	5.04	1	5.04	39.80	<0.0001	
B^2	0.089	1	0.089	0.70	0.4210	
C^2	0.70	1	0.70	5.51	0.0408	
Residual	1.27	10	0.13			
Lack of Fit	1.19	5	0.24	16.26		
Pure Error	0.073	5	0.015			
Core Total	11.26	19				

**p*-values less than 0.05 indicates model terms are significant, and values greater than 0.1 indicate the model terms are not significant. *A* = fermentation temperature, *B* = pH and *C* = inoculum concentration. Inc. and Temp stands for Inoculum concentration (% *v*/*v*) and Temperature (°C), respectively.
